# Comprehensive geriatric assessment of older patients with renal disease: a cross-sectional survey

**DOI:** 10.1038/s41598-024-59370-3

**Published:** 2024-04-16

**Authors:** Xiaoyu Chen, Yingchun Hu, Lei Peng, Hongmei Wu, Jiangwen Ren, Guanjian Liu, Li Cao, Ming Yang, Qiukui Hao

**Affiliations:** 1 Department of Nephropathy and Rheumatology Immunology, Jiulongpo People’s Hospital, Chongqing, China; 2 Department of Cardiovascular, Nanfang Hospital, Southern Medical University, Guangzhou, China; 3 Department of Medical Clinical Laboratory, People’s Hospital of Shapingba District, Chongqing, China; 4 The Center of Gerontology and Geriatrics, West China Hospital, Sichuan University, Chengdu, China; 5 Center for Evidence-Based Medicine and Clinical Epidemiology, West China Hospital, Sichuan University, Chengdu, China

**Keywords:** Kidney diseases, Health care, Nephrology

## Abstract

Multidimensional health function impairments are common in older patients with chronic kidney disease (CKD). The purpose of this study was to explore whether the risk or severity of geriatric syndrome increased with a decline in renal function. This survey was conducted for CKD patients aged  ≥ 60 years and hospitalized at West China Hospital of Sichuan University (Center of Gerontology and Geriatrics, Nephrology, and Endocrinology) and Chengdu Kangfu Kidney Disease Hospital from September 01, 2013 to June 30, 2014. Patients underwent multidimensional individualized assessments by trained doctors. Logistic regression analysis found that the risk of assisted walking (*P* = 0.001) and urinary incontinence (*P* = 0.039) increased with a decline in renal function. Regression analysis revealed that the scores of activities of daily living (*P* = 0.024), nutritional status (*P* = 0.000), total social support (*P* = 0.014), and objective support (*P* = 0.000) decreased with a decline in renal function.

## Introduction

With the advancement in social economics, living standards, and healthcare, the aging trend is prominent worldwide, and China is not an exception. The results of the seventh census in 2020 in China showed that 18.7% of the population was aged  ≥ 60 years, which was 5.44% higher than that of the 2010 census, and 13.5% of them were aged  ≥ 65 years, which was 4.63% higher than that reported in 2010^1^. The increase in the older population will undoubtedly bring great challenges to the socioeconomic development of China.

Older adults acquire more chronic illnesses than younger ones. Hypertension, diabetes, and coronary heart disease remain the primary diseases affecting the health of older adults, and these chronic diseases can lead to kidney damage. Therefore, chronic kidney disease (CKD) is increasingly common in older adults^2^. A new large cross-sectional survey in China in 2012 showed that the total prevalence of CKD in adults was 10.8%, with more female patients than male patients, and 18% and 24.2% of females were aged 60–69 years and  ≥ 70 years, respectively^3^. CKD is highly prevalent in older adults, and this leads to a heavy social and family burden and considerable medical costs. According to the 2016 data report of the China Kidney Disease Network, the total inpatient medical expenditure for patients with CKD (including end-stage renal disease) was 27.646 billion RMB. It was higher than in diabetic patients and patients without CKD^4^.

Persons with CKD often have conditions that are highly symptomatic and are multifactorial in origin^5,6^. Examples include pain, fatigue, and cognitive impairment^6^. In the geriatric literature, such multifactorial conditions have been described as “geriatric syndromes^7^”. Older adults with CKD are at high risk of geriatric syndrome, such as cognitive impairment and poor quality of life^8–11^. A French multicenter prospective cohort study showed that severe cognitive impairment increased the mortality rate and that malnutrition worsened renal dysfunction^12^, which seriously affected the quality of life in CKD patients aged  ≥ 80 years with eGFR < 45 mL/min/1.73 m^2^. Systematic review studies have suggested that depression was closely related to mortality in CKD patients, and effective control of depression could significantly reduce mortality in these patients^13^. A randomized controlled trial found that an intervention delivered outside the health encounter could have a significant (albeit modest) impact on health outcomes for patients with CKD^14^. This meant that social support could lead to good outcomes for this population. Activities of daily living (ADL) impairment was associated with increased mortality and often leaded to additional adverse outcomes^15–17^. Comprehensive geriatric assessment (CGA) is a multidisciplinary diagnostic and therapeutic process that assesses the existing health problems of older people in multiple dimensions, such as psychology, mood, and physical dysfunction. It can be used to identify multidimensional needs unique to older patients and help inform multidisciplinary, coordinated care plans to improve outcomes^8–11^. In this cross-sectional study, CGA was performed in older adults with CKD to evaluate the relationship between renal stages and geriatric syndromes.

## Results

### Sociodemographic and lifestyle characteristics

This study included 542 patients, comprising 332 (61.3%) males and 210 (38.8%) females. The median age of the participants was 76 years (60–101 years). Among the participants, 74.7% were married, and 50% had received a secondary education (including junior high school and senior high school). Approximately 36.5% of the participants were smokers, while 10.0% reported alcohol consumption.

### The distribution of geriatric syndromes and social support scores

Tables 1 and 2 was an independent risk factorshow the distribution of geriatric syndromes and social support scores. The top three geriatric syndromes with the highest occurrence rate in CKD patients were polypharmacy, risk of malnutrition and constipation.

**Table 1:** Geriatric syndromes for different stages of renal function.

	G1–G2 (n = 92)	G3a (n = 72)	G3b (n = 72)	G4 (n = 59)	G5ND (n = 48)	G5D (n = 199)	Total (n = 542)
Impaired activity of daily living, n (%)	34 (37.0)	36 (50.0)	39 (54.2)	24 (40.7)	15 (31.3)	67 (33.7)	215 (39.7)
Impaired timed up and go test, n (%)	18 (19.6)	21 (29.2)	22 (30.6)	13 (22.0)	9 (18.8)	35 (17.6)	118 (21.8)
History of fall in past 1 year, n (%)	24 (26.1)	9 (12.5)	17 (23.6)	17 (28.8)	11 (22.9)	51 (25.6)	129 (23.8)
Assisted walking, n (%)	32 (34.8)	38 (52.8)	33 (45.8)	18 (30.5)	11 (22.9)	52 (26.1)	184 (33.9)
Hearing disorder, n (%)	8 (8.7)	7 (9.7)	13 (18.1)	5 (8.5)	3 (6.3)	16 (8.0)	52 (9.6)
Visual disturbance, n (%)	9 (9.8)	4 (5.6)	7 (9.7)	3 (5.1)	6 (12.5)	20 (10.1)	49 (9.0)
Constipation, n (%)	25 (36.2)	18 (32.1)	21 (37.5)	24 (43.6)	17 (37.0)	84 (42.6)	189 (39.5)
Urinary incontinence, n (%)	15 (16.3)	14 (19.4)	5 (6.9)	11 (18.6)	7 (14.6)	17 (8.5)	69 (12.7)
Polypharmacy, n (%)	74 (80.4)	63 (87.5)	63 (87.5)	52 (88.1)	45 (93.8)	172 (86.4)	469 (86.5)
Cognition							
Mildly impaired, n (%)	25 (27.5)	28 (41.2)	21 (30.4)	17 (30.4)	16 (34.8)	81 (42.4)	188 (36.1)
Severely impaired, n (%)	24 (26.4)	12 (17.6)	18 (26.1)	10 (17.9)	6 (13.0)	27 (14.1)	97 (18.6)
Depression, n (%)	20 (22.0)	17 (24.3)	12 (16.9)	8 (13.8)	13 (27.1)	53 (26.8)	123 (22.9)
Nutritional status							
Risk of malnutrition, n (%)	38 (43.2)	25 (39.7)	26 (37.7)	24 (42.9)	28 (60.9)	107 (54.9)	248 (48.0)
Malnutrition, n (%)	10 (11.4)	6 (9.5)	8 (11.6)	10 (17.9)	5 (10.9)	39 (20.0)	78 (15.1)

G1, GFR ≥ 90 mL/min/1.73 m^2^; G2, GFR = 60–89 mL/min/1.73 m^2^; G3a, GFR = 45–59 mL/min/1.73 m^2^; G3b, GFR = 30–44 mL/min/1.73 m^2^; G4, GFR = 15–29 mL/min/1.73 m^2^; G5ND, GFR < 15 mL/min/1.73 m^2^ without dialysis; G5D, GFR < 15 mL/min/1.73 m^2^ with dialysis.

**Table 2:** Social support scores in different stages of renal function.

	G1–G2	G3a	G3b	G4	G5ND	G5D	Total
Total social support score	48.6 ± 8.2	46.7 ± 8.3	45.8 ± 7.6	47.7 ± 7.0	47.1 ± 7.1	45.6 ± 7.6	46.5 ± 7.7
Objective support score	15.0 ± 4.4	14.9 ± 4.4	15.0 ± 3.7	14.4 ± 3.8	14.2 ± 3.5	13.3 ± 3.8	14.1 ± 4.0
Subjective support score	24.7 ± 4.5	23.4 ± 4.1	22.2 ± 4.8	23.9 ± 3.7	23.5 ± 4.8	23.9 ± 4.3	23.7 ± 4.4
Support utilization score	8.9 ± 2.5	8.5 ± 2.7	8.5 ± 2.5	9.5 ± 2.0	9.6 ± 3.0	8.5 ± 2.5	8.7 ± 2.6

Data are presented as means ± standard deviations.

G1, GFR ≥ 90 mL/min/1.73 m^2^; G2, GFR = 60–89 mL/min/1.73 m^2^; G3a, GFR = 45–59 mL/min/1.73 m^2^; G3b, GFR = 30–44 mL/min/1.73 m^2^; G4, GFR = 15–29 mL/min/1.73 m^2^; G5ND, GFR < 15 mL/min/1.73 m^2^ without dialysis; G5D GFR < 15 mL/min/1.73 m^2^ with dialysis.

### Comparison of geriatric syndromes and social support scores among different stages of renal function

Abnormal gait and balance function, history of falls in the past year, assisted walking, hearing impairment, visual impairment, constipation, urinary incontinence, and polypharmacy were considered dependent variables and were assessed as binary variables (normal = 0, impaired = 1). The stages of renal function were regarded as independent variables. Logistic regression analysis results revealed that the risk of assisted walking (*P* = 0.001) and urinary incontinence (*P* = 0.039) increased with a decline in renal function (Table 3).

**Table 3:** Comparison of geriatric syndromes among the different stages of renal function (logistic regression analysis).

	G1–G2	G3a	G3b	G4	G5ND	G5D	OR	95% CI	*P*-value
Impaired timed up and go test, n (%)	18 (19.6)	21 (29.2)	22 (30.6)	13 (22.0)	9 (18.8)	35 (17.6)	1.09	0.98 − 1.21	0.120
History of fall in past one year, n (%)	24 (26.1)	9 (12.5)	17 (23.6)	17 (28.8)	11 (22.9)	51 (25.6)	0.96	0.86 − 1.06	0.377
Assisted walking, n (%)	32 (34.8)	38 (52.8)	33 (45.8)	18 (30.5)	11 (22.9)	52 (26.1)	1.18	1.07 − 1.30	0.001
Hearing disorder, n (%)	8 (8.7)	7 (9.7)	13 (18.1)	5 (8.5)	3 (6.3)	16 (8.0)	0.94	0.81 − 1.09	0.383
Visual disturbance, n (%)	9 (9.8)	4 (5.6)	7 (9.7)	3 (5.1)	6 (12.5)	20 (10.1)	1.05	0.90 − 1.23	0.528
Constipation, n (%)	25 (36.2)	18 (32.1)	21 (37.5)	24 (43.6)	17 (37.0)	84 (42.6)	0.94	0.85 − 1.03	0.178
Urinary incontinence, n (%)	15 (16.3)	14 (19.4)	5 (6.9)	11 (18.6)	7 (14.6)	17 (8.5)	1.15	1.01 − 1.31	0.039
Polypharmacy, n (%)	74 (80.4)	63 (87.5)	63 (87.5)	52 (88.1)	45 (93.8)	172 (86.4)	0.95	0.95 − 1.22	0.248

G1, GFR ≥ 90 mL/min/1.73 m^2^; G2, GFR = 60–89 mL/min/1.73 m^2^; G3a, GFR = 45–59 mL/min/1.73 m^2^; G3b, GFR = 30–44 mL/min/1.73 m^2^; G4, GFR = 15–29 mL/min/1.73 m^2^; G5ND, GFR < 15 mL/min/1.73 m^2^ without dialysis; G5D GFR < 15 mL/min/1.73 m^2^ with dialysis; OR, odds ratio; CI, confidence interval.

The ADL, cognitive function, depression, nutritional status, total social support, objective support, subjective support and support utilization scores were considered dependent variables and assessed as numerical variables. The renal function stages were considered independent variables. Regression analysis results revealed that the ADL score (*P* = 0.024), nutritional status score (*P* = 0.000), total social support score (*P* = 0.014) and objective support score (*P* = 0.000) decreased with a decline in renal function (Table 4). Only objective support in social support decreased with a decline in renal function.

**Table 4:** Comparison of geriatric syndromes and social support among different stages of renal function (regression analysis).

Dependent variable	Independent variable	b	S.E	b	t	*P*-value
ADL score	Constant term	24.19	1.17		20.71	0.000
	eGFR stage	− 0.60	0.27	− 0.10	− 2.26	0.024
Cognitive function score	Constant term	20.72	0.61		33.94	0.000
	eGFR stage	− 0.04	0.14	− 0.01	− 0.30	0.768
Depression score	Constant term	2.35	0.29		8.07	0.000
	eGFR stage	0.10	0.07	0.07	1.50	0.134
Nutritional status score	Constant term	23.60	0.44		53.13	0.000
	eGFR stage	− 0.50	0.10	− 0.21	− 5.00	0.000
Total social support score	Constant term	48.39	0.83		58.51	0.000
	eGFR stage	− 0.45	0.18	− 0.11	− 2.47	0.014
Objective support score	Constant term	15.69	0.42		37.33	0.000
	eGFR stage	− 0.38	0.09	− 0.18	− 4.07	0.000
Subjective support score	Constant term	23.71	0.47		50.05	0.000
	eGFR stage	0.00	0.10	0.00	0.01	0.996
Support utilization score	Constant term	8.90	0.28		31.96	0.000
	eGFR stage	− 0.04	0.06	− 0.03	− 0.64	0.521

ADL, activities of daily living; eGFR, estimated glomerular filtration rate; S.E, standard error.

## Discussion

CGA, a multidisciplinary approach that assesses multidimensional health indicators, can help provide an optimal treatment plan. Currently, CGA has been widely used in the older population with various diseases or health problems^18–22^. The studies showed that CGA could detect potential health problems in older adults in order to give interventions early. In this study, different stages of renal function (G1–G5, including G5ND and G5D) were comprehensively evaluated for the physical, cognition, emotional status, nutritional status, and social support aspects.

As individuals age, their ability to perform daily living activities tends to decrease. ADL impairment is associated with increased mortality and often leads to additional adverse outcomes^15–17^. It had been shown that both mild and moderate CKD were significantly associated with disability in daily activity^23,24^. Therefore, assessing ADL in CKD patients is needed as soon as possible. This study found that with the decline in renal function, the scores of ADL decreased. This study was inconsistent with previous findings. Looking forward to more relevant studies in the future.

Cognitive impairment is common in older CKD patients. This study found a relatively high occurrence rate of cognitive decline, including mild cognitive decline and severe cognitive decline, in older CKD patients, accounting for 36.1% and 18.6%, respectively, especially in dialysis patients (42.4% and 14.1%, respectively). This showed a good agreement with the findings of some previous studies^25^. In 2010, a multicenter study in the US studied the overall cognitive impairment in this population, and a positive correlation between decreased eGFR and cognitive scores was reported, i.e., the lower the eGFR, the more severe the cognitive impairment^10^. Cognitive changes occur and skills decline at different rates early in CKD. Orientation, attention, and language are particularly affected in these patients^26^. Therefore, there is a need for proactive assessment of cognitive function in older CKD patients to provide appropriate interventions.

Depression is common in the older adults and especially among patients who suffer from chronic diseases^27^. This study showed that the occurrence rate of depression was 22.9% in older CKD patients, especially end-stage patients (nearly 30%), which was consistent with many previous results^28–31^. One prospective cohort study monitored over 20 years indicated a bidirectional association between depression and CKD^32^. This study did not find a progressive increase in depression scores with decreasing renal function. But the higher the occurrence rate of depression in patients with CKD was incontestable. Studies have shown that depression was negatively associated with the patient’s quality of life and that it was an independent risk factor for death in maintenance hemodialysis patients^13,29,30,33^. Therefore, it is particularly important to evaluate depression as early as possible in older patients with CKD.

The results of this study showed that older CKD patients had a high occurrence rate of poor nutrition, especially in end-stage renal disease (G5ND: risk of malnutrition, 60.9% and malnutrition, 10.9%; G5D: risk of malnutrition, 54.9% and malnutrition, 20.0%). Thus, nutritional health problems were prevalent in older CKD patients, corroborating some previous findings^34–36^. Malnutrition not only affects the quality of life and aggravates renal dysfunction but is also an indicator of poor prognosis in older patients. Malnutrition plays an important role in the mortality-related factors of patients with end-stage renal disease. In this study, we found that nutrition status became worse as renal function declined. Hence, in older patients with CKD, providing reasonable nutritional assessment and nutritional support are important responsibilities of the medical staff.

Social support refers to positive group and family interactions. Good social support has a positive effect on the patient’s mental health^37^. It has been shown that addressing patient perception of social support could potentially improve outcomes^14,38^. This study found that social support gradually decreased in older CKD patients as the renal function declined. Therefore, for older CKD patients, we need to understand their families and their surrounding social environment and accordingly conduct health education for them and their families to enhance family support and care. For clinical workers, it is necessary to encourage the patient’s access to social activities.

Impaired mobility can be a temporary or permanent condition (it can have both physical and psychological consequences), and it can be caused by a variety of modifiable and non-modifiable risk factors^39^. This study showed that the occurrence rate of assisted walking was 33.9%, which was consistent with previous results^40^. The study found that the risk of assisted walking increased with a decline in renal function. Strategies to prevent and improve mobility limitations are strongly needed.

Due to the embarrassment associated with urinary incontinence, most patients never discuss this bothersome urinary symptom with their physicians or seek treatment^41^. This study revealed that the risk of urinary incontinence increased with a decline in renal function. At present, the cause of urinary incontinence in older CKD patients is unclear, and relevant studies are expected.

This study has several limitations. First, the analysis used a cross-sectional design. Second, Inability to establish causality. Third, subgroup sizes were relatively small, thus resulting in a lack of sufficient basis for conclusion. Despite these shortcomings, the strength of our study was that the included subjects covered all stages rather than a single stage and are more representative.

## Conclusion

In this study, we found the scores of ADL, nutritional status, total social support and objective support decreased with a decline in renal function. Meanwhile the risk of assisted walking and urinary incontinence increased with a decline in renal function. The findings of this study can provide guidance to healthcare providers to assess assisted walking, urinary incontinence, ADL, nutritional status, total social support and objective support in older patients with CKD. There is a need for further research into the underlying cause of geriatric syndromes in CKD with a view to developing therapeutic interventions. 

## Methods

### Participants

This study enrolled CKD patients aged ≥ 60 years and hospitalized at West China Hospital, Sichuan University (Center of Gerontology &Geriatrics, Nephrology, and Endocrinology) and Chengdu Kangfu Kidney Disease Hospital between September 1, 2013 and June 30, 2014. Among the 545 individuals, 3 refused investigation; thus, 542 were finally investigated, with a response rate of 99.5%.

### Inclusion criteria

The inclusion criteria were as follows: (1) age  ≥ 60 years; (2) diagnosis and staging of CKD according to the 2012 International Kidney Disease Organization “Kidney Disease: Improving Global Outcomes” guidelines^42^; and (3) CKD combined with two or more chronic diseases or geriatric syndromes, including CKD complications, as per the diagnosis recorded by the in-charge physician in the medical records.

The criteria for diagnosis and staging were as follows^42^:

1. Kidney injury (abnormal renal structure or function) for  ≥ 3 months with or without GFR decline, showing abnormal renal pathological examination (renal biopsy or imaging), abnormal blood and urine composition, and history of renal transplantation
2. GFR < 60 mL/min/1.73 m^2^ for ≥ 3 months with or without evidence of renal impairment

(GFR stages: G1, GFR ≥ 90 mL/min/1.73 m^2^; G2, GFR = 60–89 mL/min/1.73 m^2^; G3a, GFR = 45–59 mL/min/1.73 m^2^; G3b, GFR = 30–44 mL/min/1.73 m^2^; G4, GFR = 15–29 mL/min/1.73 m^2^; and G5, GFR < 15 mL/min/1.73 m^2^, including G5ND [non-dialysis] and G5D [dialysis]).

### Exclusion criteria

The exclusion criteria were as follows: (1) any acute disease, such as acute heart failure, acute kidney failure, acute liver failure, and acute respiratory failure; and (2) severe hearing or visual impairment or severe mental disorder.

### Study design

Data were collected by trained doctors during face-to-face interviews, using standardized questionnaires. At least one family member was asked to accompany the participants. When the patients could not understand unclear objective questions, the companion was allowed to answer. When subjective questions were involved, the companion was asked to avoid answering, and the participant was asked to respond personally. Informed consent was obtained from all participants or their caregivers after a thorough explanation of the study details.

### Data collection

1. General investigations.
  Baseline demographic data included sex, age (based on identity card number), marital status, education, smoking (World Health Organization [WHO] defines “continuous or cumulative smoking for ≥ 6 months in a lifetime” as smokers), and alcohol consumption (no uniform definition of alcohol consumption and drinking pattern available currently). This study defined drinking as consuming at least one standard drink per week in the past month for > 6 months. According to the WHO recommendation, one standard drink is equivalent to the amount (mL) of various types of alcoholic drinks based on the fixed pure ethanol content (10 g)^43^. The etiology of CKD was based on the final diagnosis recorded by the physician in charge in the medical records. We determined whether dialysis was performed by asking the patients and doctors.
2. Physical examinations and laboratory indicators in the last 3 months.
  Physical examinations included height (cm), weight (kg), body mass index (kg/m^2^), midpoint circumference of the upper arm (cm), and gastrocnemius muscle circumference (cm). Laboratory indicators included routine blood tests, biochemical parameters (serum albumin, glycosylated hemoglobin, parathyroid hormone, serum creatinine, serum phosphorus, and random urine albumin/creatinine ratio), and urine tests. All indicators were the results of examinations of patients conducted in secondary hospitals or higher in the last 3 months. Based on the serum creatinine level, the GFR was calculated using the Chronic Kidney Disease Epidemiology Collaboration formula, which was considered the eGFR^44^.
3. Mobility.
  Gait speed and balance were assessed using the “timed up and go” test^45,46^.
  Falls-We asked the patients if they had fallen on the ground or hit other objects (such as chairs or walls) in the past year (yes or no).
  Assisted walking- We asked the patients if they needed someone else or an aid to walk, such as walking stick, wheelchair and walking frame (yes or no).
4. Neurosensory deficits-impairment of hearing and/or vision (yes or no).
  If their hearing was satisfactory for daily conversations, watching TV, or using the phone, and if their eyesight was satisfactory for reading books or newspapers and watching TV.
5. Constipation (yes or no).
  Constipation was defined as defecation < 3 times per week without laxatives and subjective discomfort due to difficulty and/or incomplete defecation at least 25% of the time, which lasted for at least over 2 weeks.
6. Urinary incontinence (yes or no).
  The patients were asked if they had involuntary urine leakage in the past year.
7. Polypharmacy- Older people taking more than five prescribed medications were considered polypharmacy patients.
8. Activities of daily living—This study assessed the physical ADL and instrumental ADL in older patients using the Activities of Daily Living Scale developed by Lawton and Brody in 1969^47^; score < 14-patient is independent; score ≥ 16-patient is impaired. Higher score indicated worse activities of daily living.
9. Cognitive function—Saint Louis University Mental Status Examination^48^.
  High school education and above: score 27–30 normal, 20–26 mild cognitive impairment, 1–19 severe cognitive impairment.
  Lower than high school education: score 20–30 normal, 15–19 mild cognitive impairment, 1–14 severe cognitive impairment.
10. Depressive symptoms-The short version of the Geriatric Depression Scale-15^49,50^, score 0–4 normal, 5–8 mild depression, 9–11 moderate depression, 12–15 severe depression.
11. Nutrition-We used the mini nutritional assessment scale with a total of 30 points (score < 17 malnutrition, 17–23.5 risk of malnutrition,  ≥ 24 good)^51^.
12. Social support—Social support rating scale was used in the study^52^. It was consisted of three parts, which were subjective support, objective support and support utilization. The total social support score is the sum of the scores of the three parts. Higher score (individual or total) indicated more social support.

### Statistical analysis

Excel 2007 (Microsoft Excel, https://www.microsoft.com/zh-cn/microsoft-365/excel) and SPSS 17.0 (Statistical Package for the Social Sciences, https://www.ibm.com/products/spss-statistics) statistical software were used for data collation and analyses. Normally distributed measurement data are statistically described using means ± standard deviations and non-normally distributed measurement data using medians and interquartile ranges. Enumeration data are expressed as frequencies and percentages. For inter-group comparisons, the t-test and analysis of variance were used if measurement data showed normal distribution and homogeneity of variance, while the chi-square test was used for enumeration data. The linear relationship between one dependent variable and multiple independent variables was analyzed. Regression analysis was applied if the outcome variable was a numerical variable, while logistic regression analysis was applied if the outcome variable was a binary variable. The differences between groups were considered statistically significant for *P*-values ≤ 0.05.

### Institutional review board

The study was ethically approved by the Medical Ethics Committee of Sichuan University. All the procedures were performed in accordance with the relevant guidelines and regulations.

### Informed consent

Written informed consent was obtained from all the participants or the Lar legally authorized representative.

## Data Availability

Data supporting the findings of the current study are available from the first author or the corresponding author upon reasonable request.
